# Editorial

**Published:** 1874-04

**Authors:** 


### Editorial.

***The Philadelphia University of Medicine,***

Recently suppressed by an Act of the Legislature of the State of Pennsylvania, is represented in Chicago by an individual practicing medicine under the authority (?) of one of its diplomas, who, from evidence in our possession, seems to have acquired his medical education in one of the subterranean beer saloons so abundantly scattered along Milwaukee avenue, being in fact one of the category termed by Falstaff "revolted tapsters." We have been urged to expose the individual by name, which we decline to do; first, because we do not propose to advertise quacks, and secondly, because we believe that the public will profit by no teacher but experience, and its lessons must be bought, and paid for. Those who habitually place their lives in the hands of physicians of whose skill and integrity they have no evidence, have a perfect right to do so, and if their folly bears its usual fruit they must eat it, thanking themselves alone for the repast, and look to the law for their redress against these, as against other false pretenders.

***Sanitary Science.***

"*Dogberry.*—We will spare for no wit, I warrant you; here 's that [touching his forehead] shall drive some of them to a *non com.:* only get the learned writer to set down our excommunication." *Much Ado About Nothing.* Act III, Scene V.

The "excommunication" has been set down, and it runs thus: "He doubted whether syphilis could be considered a contagious disease in the strict acceptation of the word. He believed it was not contagious, inasmuch as it could not be caught in the air or by contact."

The above is a quotation from the proceedings of the Chicago Board of Health, as published in the Chicago Times, Feb. 26th, and is a sample of the sanitary science for which the city pays seventy or eighty thousand dollars per annum, and also will serve to illustrate the quality of the dead wood out of which this "board" has been constructed.

This opinion was announced during a discussion of certain propositions, the first of which was, "The principal duty and objects of sanitary science, and of boards of health, is to promote and preserve the sanitary safety and health of the citizens, and to improve their sanitary condition." If the expression of such opinions be the first step toward the fulfillment of "the principal duties of sanitary science and boards of health," then we should earnestly pray to be protected from the possible consequences of their continued fulfillment in the manner foreshadowed in the above.

Moreover, the essential correlation of "sanitary science and boards of health" implied in the same, will appear more than doubtful, through the very medium of its expression.

The operations of the Chicago Board of Health have suggested a widely different conception of its duty, its objects and its correlations than that indicated by the proposition quoted above, and a much more intimate affiliation with ward politics and garbage-contracts, than with sanitary science.

In view of the probable selection of Chicago as the place for the next meeting of the Sanitary Association of the United States, we have reason to congratulate ourselves in advance, upon the weight (avoirdupois) of authority with which local sanitary science will be sustained therein.

***Asylum for Imbeciles.***

We are told by a popular poet that, "for ways that are dark and tricks that are are vain, the heathen Chinee is peculiar," and those who have carefully watched the proceedings of the present legislature at Springfield, and have read House Bill No. 801, will see in it the finger marks of Ah Sin. This is a Bill for an "Act to establish a Board of Health for the State of Illinois." "To consist of eight (8) persons, who shall be physicians of good standing," to hold office for four years.

Section 2 of this "Bill" defines the duties of said board, in which nothing specially noteworthy occurs, except that they are to report once a year to the Governor "their *doings*, *investigations and discoveries.*"

Section 3 provides that they shall hold "regular meetings at least once every four months," that four members shall constitute a quorum; that a president shall be elected annually, *and a secretary—who shall be the executive officer of the board—quadrennially.* That "no member shall receive compensation for his services, *except the secretary;*" "Provided, also, that the actual travelling and *other expenses* incurred by any member, in performing the legitimate work of the board, be paid." That the secretary be allowed to draw upon the treasurer of the State for an amount necessary to purchase the needed stationery, and for other necessary expenses; also, "an *annual* sum for his services," "not to exceed twenty-five hundred dollars for *any one year.*"

Section 4 defines the duties of the secretary, which shall be "*to superintend* the work contemplated in this act," etc., etc.

Section 5 provides, amongst other things, "that such information and statistics," etc., "as *the board of health may deem proper*, shall be recorded," etc.

Section 6 provides for the registration of births, marriages and deaths.

Section 7 provides that all boards of health within the State shall be auxiliary to the State Board, and shall *report* annually to the *secretary* of that board at such time as he may direct.

Section 8 relates to "a seal."

Section 9 initiates the effect of the act, etc.

We said above that the finger marks of Ah Sin—whose smile was child-like and bland—could be traced in this "Bill," and a long bill it will prove to the taxpayers if it should ever become a law. We did not mean literally, because the Bill is written by some one tolerably familiar with the English language, ("which the same *he* does not understand"); but metaphorically, some good-natured friend having put his crude ideas into intelligible shape.

It has recently been stated by the secular press that no less than twenty-two boards now encumber the administration of this much-governed State, and now when the curses of the people both broad and deep are execrating the corruption growing out of these, it is coolly proposed to add another to the already too long list.

But this one is to "consist of eight persons, who shall be physicians of good standing." Does Ah Sin suppose that eight physicians in good standing are to be found, willing to accept such positions, assume the weighty responsibility incident thereto, and conduct the *work* of *investigations* and *discoveries*, defined in the 2nd section of the "Bill," without compensation? He does not suppose it, and if he does not, nobody else is idiot enough to do so. Moreover, there is no such intention or desire on the part of the framer of the Bill. The personnel of the Board will consist of one champion dead-beat (pardon the slang) who will be the secretary, and seven professional nonentities, whose duties will be limited to giving a coloring of legality to the secretary's performances, and —most onerous of all—to listen to his long winded "reports."

In view of the abominable tinkering to which the original Health-Bill, proposed for Chicago, was subjected at Springfield, resulting in the passage of the present legislative abortion; in view of the public ridicule and odium which the Board as now constituted has attracted, by its utter failure to accomplish the objects for which it was ostensibly designed, it would seem to demand a degree of modest assurance amounting to impudence, to attempt the creation of another such imposition upon the credulity and endurance of the community.

Moreover, this House-Bill No. 801, providing for a State Board of Health, is but a cautious first step in the initiation of another and bolder scheme, *i. e.*, the passage through Congress of a law authorizing the creation of a National Board of Health, ostensibly for the preservation of the public health, the cultivation of sanitary science, and the education of the people therein; but in reality to create official position for small professional politicians and placemen, too ignorant and indolent to earn for themselves position and respect in the legitimate walks of professional life.

The tendency of such institutions is to become asylums for worn-out political hacks, having as much knowledge of sanitary science as a hedge priest has of theology; until by their corruption they engender the elements of their own disorganization.

One intelligent and well educated physician, clothed with proper authority, and having the necessary assistance, could render more efficient service in the preservation of public health, the advancement of sanitary science, and the education of the people therein, than this proposed State Board and all its auxiliaries combined. But he must be both intelligent and well educated, and not simply a literary and scientific junk shop, crammed with odds and ends of second-hand information and worn-out ideas borrowed from popular treatises on "Science made easy," or picked up in casual conversations with educated men.

### *Abstract of the Proceedings of the "Chicago Society of Physicians and Surgeons," for the year 1873.*

### I. Reports.

*a.* Annual Report of Section on Practice of Medicine—one paper.

*b.* Annual Report of Section on Surgery—three papers.

1. Strictures of Urethra and Rectum.*
2. Electro-Therapeutics of Surgery.
3. Staphyloraphy and Uranoplasty, with exhibition of gag and models.*

*c.* Annual Report of Section on Pathology—two papers.

1. Pathology of Nervous System.*
2. Pathology of Ovarian Tumors.

*d.* Annual Report of Section on Obstetrics and Diseases of Women and Children—two papers.

1. Diseases of Women.*
2. Diseases of Children.

*e.* Annual Reports of Officers of the Society.

*f.* Report on the Literature of Cholera, with Abstracts from the Later Authorities.*

*g.* Report of Committee on the Prophylaxis and Treatment of the Cholera Endemic in 1873.*

*h.* Report on the Therapeutic Value of Cosmoline.

### II. Papers.

*a.* On Centripetal and Centrifugal Neuroses.

*b.* On Pathogenesis by the use of Tobacco.*

*c.* On the Relations of the Integumentary and Generative Organs.*

*d.* On the use of Lime Vapor in Pseudo-Membranous Croup.*

*e.* On the Cervix Uteri Before, During, and After Labor. Illustrated by drawings.*

*f.* On the Genetic Relations of Marsh Fungi to Malarial Disease. Illustrated by colored sketches and microscopical preparations of palmellæ blood containing sporules, saliva and sporules, etc.*

*g.* Review of Hall's Translation of Lanfranc's Surgery, published in London in 1565.*

*h.* On the Physiological Relations of Alcohol.*

*i.* On some Questions in Therapeutics.

*j.* On the Waxy Kidney.*

### III. Reports of Cases.

*a.* Cases of Cholera, occurring near the Southern Limits of the City of Chicago —Endemic of 1873.

*b.* Additional Cases in same general place and time. Illustrated by map of locality.

*c.* Additional Cases in the same locality, occurring later in the season.

*d.* Cases of Cholera in Louisville, occurring in 1856.

*e.* Twelve Cases of Cholera in the City of Chicago in 1873.*

*f.* Case of Criminal Abortion—fatal from hemorrhage.

*g.* Cases of Intra-laryngeal and Tracheal Medication.

*h.* Case of Chronic Inflammation of the Stomach, simulating Cancer, with Report of Necroscopy and Microscopic Pathology, and exhibition of stomach in gross, with magnified and illuminated sections.*

*i.* Cases of Uterine Fibroids relieved by the Hypodermic Injection of Ergotine.

*j.* Case of Foreign Body in the Urethra.

*k.* Case of Gunshot Injury of the Cerebral Sinuses, terminating fatally.

*l.* Case of Intense Sciatic Pain from Suppressed Menstruation, relieved by the Thermo-Electric Bath.

*m.* Case of Syphilitic Destruction of the Epiglottis, with unimpaired Power of Deglutition. Exhibition of patient.

### IV. Specimens Presented.

*a.* Semi-calcareous tumor, removed from the arm of a pregnant female—and report of case.

*b.* Monster; with complete visceral ectrophy, four mammary glands, and no genito-urinary nor intestinal apertures.

*c.* Heart; ruptured from muscular degeneration— and report of case.

*d.* Right auricle; perforated by pistol ball—and report of case.

*e.* Ossified cardiac valves—and report of case.

*f.* Cancer of uterus—and report of case.

*g.* Rice water dejections in cholera, mounted on microscopic slides, and illuminated—with reports from cases

V. Exhibitions.

*a.* Anatomical models of the Chicago University. Recently purchased in Germany.

*b.* Reproductions in colored crayon, of photographs of cutaneous diseases.

*c.* Models of invalid and operating chairs, close stools, etc.

*d.* Spectroscopic and other scientific apparatus.

| | |
|---|---|
| Number of meetings in 1873 | 22 |
| Total attendance | 436 |
| Average attendance each meeting | 19.8 |

☞ Papers marked with a star, have been published in the medical journals of the city.

